# Manifestations of classical physics in the quantum evolution of correlated spin states in pulsed NMR experiments

**DOI:** 10.1002/cmr.a.21398

**Published:** 2017-10-10

**Authors:** Martin Ligare

**Affiliations:** ^1^ Department of Physics and Astronomy Bucknell University Lewisburg PA USA

**Keywords:** classical physics, correlated spins, COSY, magnetic resonance, product operators

## Abstract

Multiple‐pulse NMR experiments are a powerful tool for the investigation of molecules with coupled nuclear spins. The product operator formalism provides a way to understand the quantum evolution of an ensemble of weakly coupled spins in such experiments using some of the more intuitive concepts of classical physics and semi‐classical vector representations. In this paper I present a new way in which to interpret the quantum evolution of an ensemble of spins. I recast the quantum problem in terms of mixtures of pure states of two spins whose expectation values evolve identically to those of classical moments. Pictorial representations of these classically evolving states provide a way to calculate the time evolution of ensembles of weakly coupled spins without the full machinery of quantum mechanics, offering insight to anyone who understands precession of magnetic moments in magnetic fields.

## 1 INTRODUCTION

The past several decades have seen an explosion in the development of new and powerful experimental techniques investigating coupled nuclei using multiple‐pulse NMR experiments. The sophistication of these techniques presents a pedagogical problem: How can these techniques be introduced to students and practitioners who may never have the opportunity to develop a deep understanding of the quantum mechanical formalism that provides a quantitative description of all aspects of the dynamics of coupled spins? In this paper I identify classically evolving states in a fully quantum mechanical treatment of the time evolution of coupled spins in multiple‐pulse NMR experiments. Pictorial representations of these quantum states have the potential to provide physical insight to anyone who understands classical precession of moments in magnetic fields.

The challenge of reconciling quantum and classical pictures of magnetic resonance goes back to the early days of NMR. Purcell (at Harvard) and Bloch (at Stanford) had very different perspectives on NMR—differences so great that their respective achievements were termed “the two discoveries of NMR” in an excellent review reconciling the quantum and classical points of view of single‐spin NMR.^1^ It is now understood that classical physics, with accompanying pictorial representations of vector moments, is sufficient to describe and understand the time evolution of quantum expectation values, and thus the results of all NMR experiments involving ensembles of single spins.^2^, ^3^ The correspondence between classical and quantum results for precessing spins has also been discussed in terms of Ehrenfest's theorem in several undergraduate texts.^4^, ^5^ Identifying classical descriptions and representations of NMR experiments on coupled spins has proved a more difficult challenge. Quantum mechanics provides, of course, a complete description of all experiments, but quantum mechanics is not intuitive for those not well versed in the formalism. The product operator method was introduced in 1983^6^, ^7^, ^8^ with the express intent of providing a “middle course” between the full quantum mechanical density matrix theory and the more intuitive classical and semiclasical vector models. In the ensuing years many authors have proposed a variety of pictorial models to help students navigate this “middle course” between classical and quantum mechanical perspectives (see, for example,^9^, ^10^, ^11^, ^12^, ^13^, ^14^).

In this paper, I present a new “middle course.” Like the product operator formalism, my “course” is fully quantum mechanical, but it is based on the identification of pure quantum states of two spins that evolve in ways that have *exact* analogs in the evolution of classical magnetic moments. As I show, the terms in the product operator expansion of the density matrix (which do not evolve analogously to classical moments) can all be associated with mixtures of two of these classically evolving pure quantum states. The pure quantum states I introduce are easy to represent pictorially, and these representations provide an easy way to determine the exact density matrix for ensembles of weakly coupled spins in pulsed NMR experiments.

The formalism I introduce is not intended to replace product operators as a tool for analyzing experiments on coupled spins. Rather, it is intended to provide a new perspective, informed by classical physics, on the evolution of the terms in the product operator expansion of the density matrix. The tools discussed in this paper may also find use in introducing multiple‐pulse NMR experiments to those without a strong background in quantum mechanics.

## 2 CLASSICALLY EVOLVING QUANTUM STATES OF SINGLE SPINS

### 2.1 Pictorial representations of general single spin states

In this section I consider individual spin‐1/2 nuclei. The nuclei have a magnetic moment **m** which is proportional to the angular momentum, ie, **m** = γ**I**, where **I** is the nuclear angular momentum vector and γ is the gyromagnetic ratio. In a constant magnetic field, **B**, the moments experience a torque

(1) τ=m×B,

and precess around the axis of the field; if the field points in the +*z* direction in some coordinate system, the moments precess in a clockwise sense (when looking down from the +*z* direction), and the magnitude of the angular frequency is ω^0^ = γ*B*, so that the azimuthal angle of the moment's direction is given by φ = φ_0_ − ω^0^ *t*. The energy associated with the orientation of a moment is

(2) E=−m·B.

In quantum discussions of magnetic resonance, it is conventional to use as a basis the “spin‐up” and “spin‐down” states |+*z*〉 and |−*z*〉 (often labeled as |α〉 and |β〉, respectively). When the magnetic field points along the *z*‐axis, these states are eigenstates of the operator ${\hat{I}}_{z}$ corresponding to the observable *z*‐component of angular momentum, with eigenvalues 1/2 and −1/2, respectively. (Quantum angular momentum operators will be defined without the factor ℏ.) These states are simultaneously eigenstates of the Hamiltonian with energy eigenvalues −ℏω^0^/2 and +ℏω^0^/2. I will use this conventional basis for all matrix representations of operators and states in this paper. (Operators will be designated with a “hat,” and matrix representations of operators without one.)

Outcomes of measurements made on spins are governed by the rules of quantum mechanics, and therefore no classical pictorial representation can completely represent all aspects of a spin state. On their own, the basis states |±*z*〉 do not display many aspects of the general classical behavior of spins. Measurements of the *z*‐component of angular momentum always give the same value, but measurements of any component in the *x*‐*y* plane give random results of ±ℏ/2. There is no sense in which moments in these basis states exhibit precession. States with behavior that corresponds more closely to classical expectations, however, are well‐known and easy to construct. These states are discussed in many magnetic resonance texts and papers (see, for example^2^, ^3^, ^15^), and the general mapping of the dynamics of two‐state quantum systems to the geometry of 3‐space was elucidated by Feynman, et al.^16^ I will briefly summarize the results that can be found in the cited texts (as well as many other texts and articles).

Consider a magnetic moment in the linear combination state

(3) $$\begin{array}{cc} \left|\theta ,\phi \right\rangle & =\text{cos}\frac{\theta }{2}\;|+z\rangle +e^{i\phi }\text{sin}\frac{\theta }{2}\left|-z\right\rangle \\ & ⟶\left(\begin{array}{c} \text{cos}\frac{\theta }{2}\\ e^{i\phi }\text{sin}\frac{\theta }{2} \end{array}\right), \end{array}$$

where θ and φ correspond to conventional angular spherical coordinates in real 3‐space. Measurement of the component of angular momentum along an axis defined by the angles θ and φ will result in a value +ℏ/2 with a probability of 1. In other words, this is an eigenstate of the operator

(4) $${\hat{I}}_{\theta ,\phi }=\text{sin}\theta \text{cos}\phi \;{\hat{I}}_{x}+\text{sin}\theta \text{sin}\phi \;{\hat{I}}_{y}+\text{cos}\theta \;{\hat{I}}_{z}.$$

**Figure 1:**
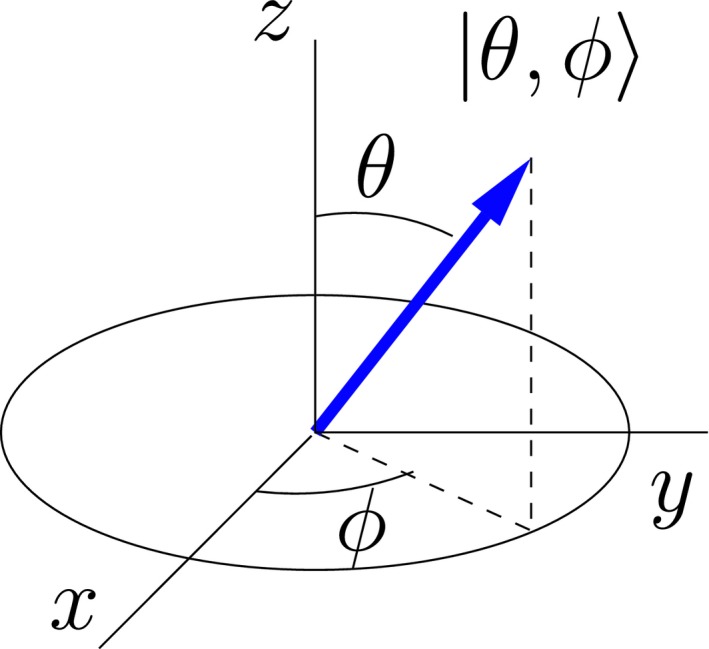
Arrow representation of the spin state $\left|\theta ;\phi \right\rangle =\text{cos}\frac{\theta }{2}\;|+z\rangle +e^{i\phi }\text{sin}\frac{\theta }{2}\;\left|-z\right\rangle$. Measurement of the component of the angular momentum along the direction of the arrow gives the value +ℏ/2 with probability 1

The state |θ, φ〉 can be represented pictorially by an arrow along the direction given by the angles θ and φ, as in Figure 1. In this “arrow representation,” the spin‐up state |+*z*〉 is represented by an arrow pointing in the +*z* direction, and the spin‐down state |−*z*〉 by an arrow pointing in the −*z* direction. The state with “spin‐up” along the *x*‐axis, given by the linear combination of the |+*z*〉 and |−*z*〉 states

(5) $$\begin{array}{c} |+x\rangle =|\theta \;=\;\pi /2,\phi \;=\;0\rangle =\frac{1}{\sqrt{2}}\left(|+z\rangle +|-z\rangle \right)\to \frac{1}{\sqrt{2}}\left(\begin{array}{c} 1\\ 1 \end{array}\right), \end{array}$$

is represented by an arrow along the +*x*‐axis, and the general state given in Equation (3) is represented by an arrow in the direction given by θ and φ.

The results of measurements of angular momentum components that do not lie along the axis of an “arrow” can *not* be predicted by taking classical geometric projections of the arrow onto other directions; such measurements will always yield random values of +ℏ/2 and −ℏ/2. The arrows display information about the probabilities of obtaining +ℏ/2 or −ℏ/2 for any measurement, but they can *not* be regarded as classical vectors when predicting results of individual spin measurements. The arrows do, however, evolve in time identically to classical magnetic moment vectors, as I will discuss in the next section.

It should be noted that the arrows used in this representation are not the same as the arrows representing magnetization vectors in semi‐classical representations of magnetic resonance, like the well‐known Bloch vectors. As a simple example, consider a 50:50 mixture of spin‐up and spin‐down nuclei. Although the magnetization vectors of the two spin states cancel, the density matrix is not zero. The information about the density matrix is retained in the representation with two arrows, with appropriate weighting factors. In addition, the arrows in the “arrow representation” do not decompose into components along the axes.

### 2.2 Evolution of single‐spin states in magnetic fields

The advantage of the “arrow representation” of quantum spin states becomes apparent when the spin is placed in a magnetic field. The quantum spin state evolves in such a way that the arrow representing the state precesses exactly as a classical magnetic moment with the same orientation. For example, in a constant magnetic field in the +*z* direction, the arrow representing the state |+x⟩=|θ=π/2,ϕ=0⟩ (and pointing in the +*x* direction) will precess in the *x*‐*y* plane as the state evolves into |θ=π/2,ϕ=−γt⟩.

In contrast, the spin‐up state |+*z*〉, represented by a arrow pointing along the direction of the field, is a stationary quantum state and does not precess, just as a classical moment aligned with a field does not precess. Classical physics provides a complete description of the evolution of individual quantum spin states; it also describes the time‐dependent quantum expectation values of observables for ensembles of spins. It is only when measurements made on individual spins are considered that classical and quantum predictions differ.

## 3 REPRESENTING PURE QUANTUM STATES OF SPIN PAIRS

### 3.1 Basis states for weakly coupled spin pairs

For weakly interacting inequivalent spins in an isotropic liquid phase, in a magnetic field with magnitude *B*_0_ pointing in +*z* direction, the approximate Hamiltonian is^2^

(6) $$\hat{H}≃-\hbar \omega_{1}^{0}\;{\hat{I}}_{1z}-\hbar \omega_{2}^{0}\;{\hat{I}}_{2z}+2\pi \hbar J\;{\hat{I}}_{1z}{\hat{I}}_{2z},$$

where $\omega_{1}^{0}=\gamma_{1}B_{0}$, $\omega_{2}^{0}=\gamma_{2}B_{0}$, and the constant scalar *J* characterizes strength of the coupling between the spins.

The conventional basis for two spins consists of the four states |α, α〉 = |+*z*; +*z*〉, |α, β〉 = |+*z*; −*z*〉, |β, α〉 = |−*z*; +*z*〉, and |β, β〉 = |−*z*, −*z*〉. These states are eigenstates of the Hamiltonian, with energies

(7a) $$E_{+z,+z}=\frac{\hbar }{2}\left(-\omega_{1}^{0}-\omega_{2}^{0}+\pi J\right)$$

(7b) $$E_{+z,-z}=\frac{\hbar }{2}\left(-\omega_{1}^{0}+\omega_{2}^{0}-\pi J\right)$$

(7c) $$E_{-z,+z}=\frac{\hbar }{2}\left(\omega_{1}^{0}-\omega_{2}^{0}-\pi J\right)$$

(7d) $$E_{-z,-z}=\frac{\hbar }{2}\left(\omega_{1}^{0}+\omega_{2}^{0}+\pi J\right).$$

As in the case of a single spin, these energy eigenstates do not exhibit precession around the fields in the *z*‐direction.

### 3.2 Pictorial representation of pure states of spin pairs

In this section I extend the arrow representation appropriate for single spins and introduce a double‐arrow representation to cover cases of two correlated spins. These states are direct products of two single‐spin states like those detailed in Equation (3).

**Figure 2:**
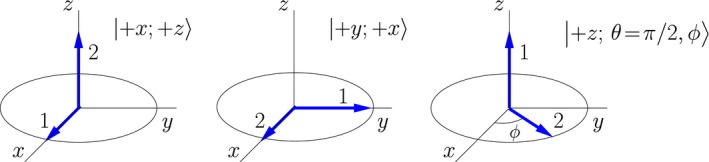
Examples of double‐arrow representations of states of two correlated spins. The arrows give directions for which simultaneous measurements of angular momentum components will both yield +ℏ/2 with probability 1 [Color figure can be viewed at wileyonlinelibrary.com]

As a first example, consider the two‐spin states represented in Figure 2. The arrows in this figure represent the two directions for which the simultaneous measurement of the component of angular momentum for each single spin are both certain to yield +ℏ/2. For the two‐spin state on the left, simultaneous measurement of the *x*‐component of angular momentum of spin 1 and the *z*‐component of angular momentum spin 2 are both certain to yield values of +ℏ/2; I label this state |+*x*; +*z*〉 ≡ |+*x*〉_1_ ⊗ |+*z*〉_2_. The two‐spin state in the middle yields a similar result for measurement of the *y*‐component of spin 1 and the *x*‐component for spin 2: both component measurements are certain to yield values of + ℏ/2. The double‐arrow states are not limited to components along Cartesian axes. An example in which one of the angular momentum components is measured along an arbitrary axis in the *x*‐*y* plane is illustrated on the right in Figure 2.

These double‐arrow states can be written in terms of the standard basis of projections along the *z*‐axis. For example, the state on the left in Figure 2 can be written

(8) $$\begin{array}{cc} \left|+x;+z\right\rangle & \equiv {\left|+x\right\rangle }_{1}⊗{\left|+z\right\rangle }_{2}=\frac{1}{\sqrt{2}}\left({\left|+z\right\rangle }_{1}+{\left|-z\right\rangle }_{1}\right)⊗{\left|+z\right\rangle }_{2}\\ & =\frac{1}{\sqrt{2}}\left(|+z;+z\rangle +|-z;+z\rangle \right)⟶\frac{1}{\sqrt{2}}\left(\begin{array}{c} 1\\ 0\\ 1\\ 0 \end{array}\right). \end{array}$$

More generally, two‐spin states can be written

(9) $$\begin{array}{cc} |\theta_{A},\phi_{A};\theta_{B},\phi_{B}\rangle & \equiv |\theta_{A},\phi_{A}\rangle {}_{A}⊗\left|\theta_{B},\phi_{B}\right\rangle {}_{B}\\ & =\;\left(\cos\frac{\theta_{A}}{2}{\left|+z\right\rangle }_{A}+{\text{e}}^{i\phi_{A}}\sin\frac{\theta_{A}}{2}\;{\left|-z\right\rangle }_{A}\right)\\ & \;⊗\left(\cos\frac{\theta_{B}}{2}{\left|+z\right\rangle }_{B}+{\text{e}}^{i\phi_{B}}\sin\frac{\theta_{B}}{2}\;{\left|-z\right\rangle }_{B}\right)\\ & =\;\left(\cos\frac{\theta_{A}}{2}\cos\frac{\theta_{B}}{2}\right)\left|+z;+z\right\rangle \\ & \;+\left({\text{e}}^{i\phi_{B}}\cos\frac{\theta_{A}}{2}\sin\frac{\theta_{B}}{2}\right)\left|+z;-z\right\rangle \\ & \;+\left({\text{e}}^{i\phi_{A}}\sin\frac{\theta_{A}}{2}\cos\frac{\theta_{B}}{2}\right)\left|-z;+z\right\rangle \\ & \;+\left({\text{e}}^{i(\phi_{A}+\phi_{B})}\sin\frac{\theta_{A}}{2}\sin\frac{\theta_{B}}{2}\right)\left|-z;-z\right\rangle \\ & \;⟶\left(\begin{array}{c} \cos\frac{\theta_{A}}{2}\cos\frac{\theta_{B}}{2}\\ {\text{e}}^{i\phi_{B}}\cos\frac{\theta_{A}}{2}\sin\frac{\theta_{B}}{2}\\ {\text{e}}^{i\phi_{A}}\sin\frac{\theta_{A}}{2}\cos\frac{\theta_{B}}{2}\\ {\text{e}}^{i(\phi_{A}+\phi_{B})}\sin\frac{\theta_{A}}{2}\sin\frac{\theta_{B}}{2} \end{array}\right). \end{array}$$

## 4 TIME EVOLUTION OF DOUBLE‐ARROW STATES

The time evolution of double‐arrow pure states is straightforward to deduce from the simple time dependence of the eigenstates of the Hamiltonian given in Equation (6); the details for a general double‐arrow state are given in the “Appendix.” In some special cases the quantum double‐arrow states remain in a factored form as the product of two spin states that both evolve in a simple manner. These special‐case states are the subject of this section, and will be the states used in the analysis of a multi‐pulse experiment. It should be noted that the classical analogs of these special case quantum states also exhibit simple time evolution. Conversely, the quantum states that do not factor in this manner correspond to those classical configurations with coupling that leads complicated classical time evolution.

### 4.1 Evolution of uncoupled spin pairs in magnetic fields

For uncoupled spins, the quantum mechanical time evolution of the double‐arrow states in constant magnetic fields is easy to visualize, because each of the single‐arrow states making up the double‐arrow evolves classically and independently. (The factoring of these quantum states is illustrated in the Appendix.) For example, in a constant field in the +*z* direction with magnitude *B*_0_, the state |+*x*, +*z*〉 illustrated in Figure 2 precesses clock‐wise (as viewed from the +*z*‐axis) at a rate $\omega_{1}^{0}=\gamma_{1}B_{0}$, evolving into another double‐arrow state:

(10) $$\left|\psi \left(0\right)\right\rangle =\left|+x;+z\right\rangle ⟶\left|\psi \left(t\right)\right\rangle =|\theta_{1}\;=\;\pi /2,\phi_{1}=-\omega_{1}^{0}t\;;\;+z〉,$$

with the tip of the arrow corresponding to spin 1 tracing out a circle in the *x*‐*y* plane. In a frame rotating clockwise around the *z*‐axis with angular frequency ω_ref_, the tip of the arrow precesses at the offset frequency $\Omega_{1}^{0}=\omega_{1}^{0}-\omega_{\mathrm{ref}}$. For the state in the middle in Figure 2, each of the arrows precesses at its own rate, giving (in the rotating frame)

(11) $$\begin{array}{c} \left|\psi \left(0\right)\rangle =|+y;+x\rangle \to |\psi \left(t\right)\rangle =\right|\theta_{1}\;=\;\pi /2,\\ \;\phi_{1}=\;\pi /2-\Omega_{1}^{0}t\;;\;\theta_{2}\;=\;\pi /2,\phi_{2}\;=\;-\Omega_{2}^{0}t\rangle , \end{array}$$

**Figure 3:**
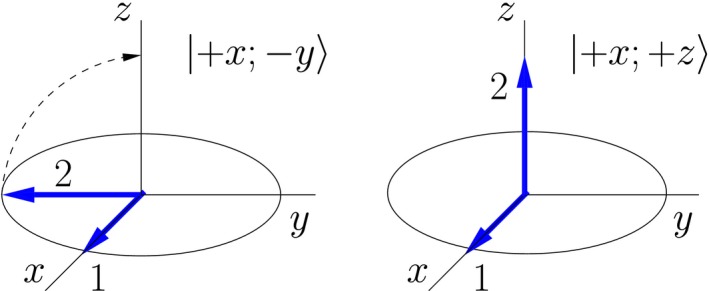
The effect of a (π/2)_*x*_ pulse on a double‐arrow state of two spins [Color figure can be viewed at wileyonlinelibrary.com]

The same kind of evolution also occurs during strong rf pulses, as is illustrated in Figure 3. In the hard pulse approximation, a (π/2)_*x*_ pulse applied to the state |+*x*; −*y*〉 rotates the arrow for spin 2 to the *z*‐axis, while leaving the arrow for spin 1 pointing in the +*x* direction, resulting in the spin‐pair state |+*x*; +*z*〉.

### 4.2 Evolution of double‐arrow states of coupled spin pairs in a magnetic field

**Figure 4:**
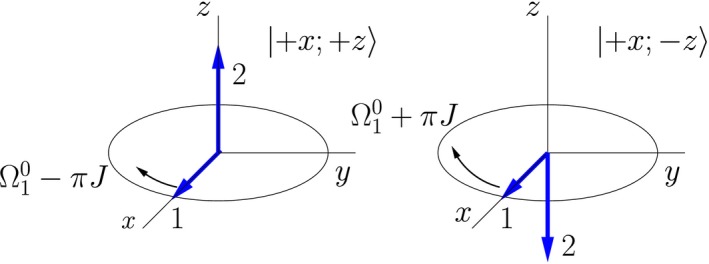
Time evolution (in the rotating frame) of a double‐arrow state of two weakly coupled spins in an external field. The precession rate of moment 1 depends on the orientation of moment 2 [Color figure can be viewed at wileyonlinelibrary.com]

For weakly coupled spins pairs the time evolution of a system starting in a double‐arrow state is in general more complex than the simple precession discussed in the previous section, but it simplifies if one of the spins is aligned (or anti‐aligned) with the external magnetic field. (This is true for classical spins as well, because an aligned moment does not precess, resulting in a constant field due to the moment.) When one of the arrows is aligned with the field, the other arrow simply precesses around the field, and the spin pair remains in a double‐arrow state as it evolves. The angular frequency of the precessing moment depends on the orientation of the stationary moment with respect to the field. An example of this is illustrated in Figure 4. A more detailed discussion of this orientation dependence is contained in the Appendix.

## 5 DOUBLE‐ARROW STATES AND PRODUCT OPERATORS

### 5.1 Product operators as mixtures of pure states

**Figure 5:**
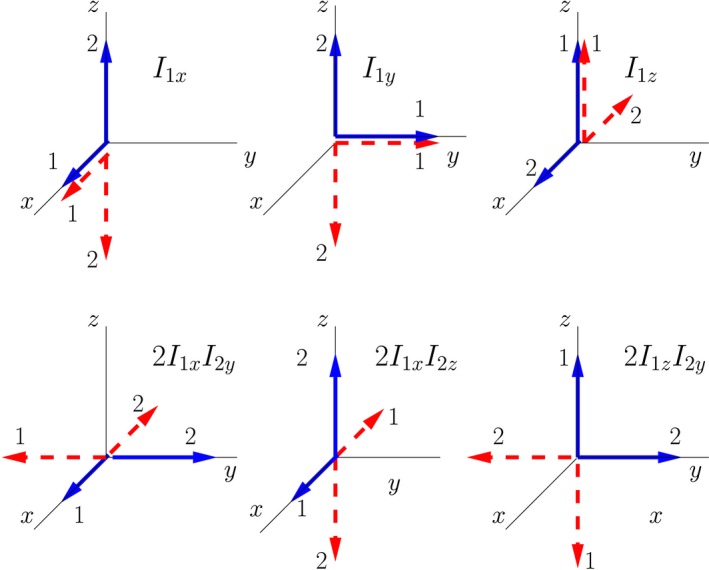
Mixtures of double‐arrow states that correspond to representative “product operators.” The solid blue lines represent a single pure quantum state of a spin‐pair, and the dotted red lines represents a different single pure state [Color figure can be viewed at wileyonlinelibrary.com]

The double‐arrow states I have introduced have a close relationship with what are known as product operators.^6^, ^7^, ^8^ The product operator formalism provides a decomposition of the density matrix of an ensemble of spins into terms that are orthogonal (with respect to the trace), and that evolve in easy‐to‐characterize ways. (I find the name “product operator,” and the labeling of the terms with the same symbols used for angular momentum, to be somewhat misleading. Each term in the product operator expansion is simply a density matrix for a specific ensemble mixture. The terms do not correspond to dynamical operators that appear in the Hamiltonian. In this paper, “product operator” will always refer to a specific density matrix, not a dynamcial operator.) The terms in a product operator expansion are not density matrices of pure states. As I will demonstrate, however, each of the terms in the product operator expansion is related to the density matrix of a 50:50 mixture of two classically evolving double‐arrow states. The correspondences between representative product operators and mixtures of spin‐pair double‐arrow states are illustrated in Figure 5, and will be discussed below.

As an example of how this association works, consider the term in the expansion of the density matrix labeled *I*_1*z*_. This term corresponds to the density matrix

(12) $$I_{1z}=\frac{1}{2}\left(\begin{array}{cccc} 1 & 0 & 0 & 0\\ 0 & 1 & 0 & 0\\ 0 & 0 & -1 & 0\\ 0 & 0 & 0 & -1 \end{array}\right).$$

A non‐zero *I*_1*z*_ term implies a net polarization of nucleus 1 along the *z*‐axis, with no preferred orientation for nucleus 2. The phrase “no preferred orientation” is an inherently classical expression; the spins labeled 2 must be in some quantum state, and all pure quantum spin states exhibit a polarization in some direction. The phrase “no preferred direction” implies that the ensemble of spins is in a mixture of states, and the mixture must be consistent with the isotropic distribution of results for measurements of the components of angular momentum of spin 2. A 50:50 mixture of two appropriately chosen double‐arrow states satisfies these criteria.

As one example, consider a 50:50 mixture of the double‐arrow states

(13) $$\left|+z;+y\right\rangle ⟶\frac{1}{\sqrt{2}}\left(\begin{array}{c} 1\\ i\\ 0\\ 0 \end{array}\right)\;\text{and}\;\left|+z;-y\right\rangle ⟶\frac{1}{\sqrt{2}}\left(\begin{array}{c} 1\\ -i\\ 0\\ 0 \end{array}\right),$$

with associated density matrices

(14) $$\begin{array}{cc} \rho_{+z,+y} & =\frac{1}{2}\left(\begin{array}{cccc} 1 & -i & 0 & 0\\ i & 1 & 0 & 0\\ 0 & 0 & 0 & 0\\ 0 & 0 & 0 & 0 \end{array}\right)\;\text{and}\;\\ \rho_{+z,-y} & =\frac{1}{2}\left(\begin{array}{cccc} 1 & i & 0 & 0\\ -i & 1 & 0 & 0\\ 0 & 0 & 0 & 0\\ 0 & 0 & 0 & 0 \end{array}\right). \end{array}$$

The density matrix for a 50:50 mixture of these two pure states is

(15) $$\frac{1}{2}\;\rho_{+z,+y}+\frac{1}{2}\;\rho_{+z,-y}=\frac{1}{2}\left(\begin{array}{cccc} 1 & 0 & 0 & 0\\ 0 & 1 & 0 & 0\\ 0 & 0 & 0 & 0\\ 0 & 0 & 0 & 0 \end{array}\right).$$

The expectation value for any component of angular momentum of nucleus 2 is zero, ie, Tr(ρ*I*_2*j*_) = 0, for any component *j*. This means that there is no preferred orientation for nucleus 2 in the ensemble of spin states. The expectation value of the *z*‐component of angular momentum of nucleus 1 is ℏ/2 × Tr(ρ*I*_1*z*_) = ℏ/2, as desired.

Comparing Equations (12) and (15) gives the relationship between the product operator *I*_1*z*_ and the density matrix for a mixture of the two double‐arrow pure states:

(16) $$\frac{1}{2}\left(\rho_{+z,+y}-\frac{1}{4}\;E\right)+\frac{1}{2}\left(\rho_{+z,-y}-\frac{1}{4}\;E\right)=\frac{1}{2}\;I_{1z},$$

where E is the identity matrix. The product operator is simply twice the density matrix of a 50:50 mixture of double‐arrow states, with a term proportional to the identify matrix subtracted to make the product operator mixture traceless.

Similar considerations give the general results

(17a) $$\frac{1}{2}\left(\rho_{+j,+k}-\frac{1}{4}\;E\right)+\frac{1}{2}\left(\rho_{+j,-k}-\frac{1}{4}\;E\right)=\frac{1}{2}I_{1j}$$

(17b) $$\frac{1}{2}\left(\rho_{+k,+j}-\frac{1}{4}\;E\right)+\frac{1}{2}\left(\rho_{-k,+j}-\frac{1}{4}\;E\right)=\frac{1}{2}I_{2j},$$

where *j* and *k* indicate directions in space. In words, a single‐spin product operator is associated with a mixture of two double‐arrow states, with one arrow from each of the states pointing along the direction specified by the product operator, and the other arrows opposite to each other along any arbitrary direction.

Note that the choice to use *y*‐components for spin 2 in the 50:50 mixture of Equation (16) is at this point arbitrary; components along any opposing directions would work just as well, giving the same result for the density matrix. Similar arbitrariness will arise in the representation of other terms in the product operator expansion too. In Section 7, where I discuss a multiple‐pulse experiment, I will make choices from the set the possible representations that make the time evolution the simplest, matching that expected for similarly aligned classical moments.

As an example of a two‐spin product operator, consider the product operator labeled 2*I*_1*x*_ *I*_2*y*_, corresponding to the density matrix

(18) $$2I_{1x}I_{2y}=\frac{1}{2}\left(\begin{array}{cccc} 0 & 0 & 0 & -i\\ 0 & 0 & i & 0\\ 0 & -i & 0 & 0\\ i & 0 & 0 & 0 \end{array}\right).$$

A density matrix expansion with a non‐zero 2*I*_1*x*_ *I*_2*y*_ term implies that measurements of the *x*‐component of spin 1 and the *y*‐component of spin 2 will be correlated, ie, a measurement yielding a positive value of the *x*‐component of angular momentum of nucleus 1 is more likely than not to be accompanied by a positive *y*‐component of the angular momentum of nucleus 2, and measurement of a negative value of the *x*‐component of angular momentum of nucleus 1 is more likely than not to be accompanied by a negative *y*‐component of the angular momentum of nucleus 2.

Consider the 50:50 mixture of the double‐arrow states in which the correlation of the *x‐* and *y*‐components of the spins is manifest:

(19) $$\left|+x;+y\right\rangle ⟶\frac{1}{\sqrt{2}}\left(\begin{array}{c} 1\\ i\\ 1\\ i \end{array}\right)\;\text{and}\;\left|-x;-y\right\rangle ⟶\frac{1}{\sqrt{2}}\left(\begin{array}{c} 1\\ -i\\ -1\\ 1 \end{array}\right),$$

with associated density matrices

(20) $$\begin{array}{cc} \rho_{+x,+y} & =\frac{1}{4}\left(\begin{array}{cccc} 1 & -i & 1 & -i\\ i & 1 & i & 1\\ 1 & -i & 1 & -i\\ i & 1 & i & 1 \end{array}\right)\;\text{and}\\ \rho_{-x,-y} & =\frac{1}{4}\left(\begin{array}{cccc} 1 & i & -1 & -i\\ -i & 1 & i & -1\\ -1 & -i & 1 & i\\ i & -1 & -i & 1 \end{array}\right). \end{array}$$

The density matrix for a 50:50 mixture of these two pure states is

(21) $$\frac{1}{2}\;\rho_{+x,+y}+\frac{1}{2}\;\rho_{-x,-y}=\frac{1}{4}\left(\begin{array}{cccc} 1 & 0 & 0 & -i\\ 0 & 1 & i & 0\\ 0 & -i & 1 & 0\\ i & 0 & 0 & 1 \end{array}\right).$$

Comparing Equations (18) and (21) gives the relationship between the product operator 2*I*_1*x*_ *I*_2*y*_ and the density matrix for a mixture of two double‐arrow pure states:

(22) $$\frac{1}{2}\left(\rho_{+x,+y}-\frac{1}{4}\;E\right)+\frac{1}{2}\left(\rho_{-x,-y}-\frac{1}{4}\;E\right)=\frac{1}{2}\left(2I_{1x}I_{2y}\right).$$

Once again, the product operator is simply twice the density matrix of a mixture of symmetrically oriented double‐arrow states (with a term proportional to the identity matrix subtracted).

More generally, for a two‐spin product operator term we have

(23) $$\frac{1}{2}\left(\rho_{+j,+k}-\frac{1}{4}\;E\right)+\frac{1}{2}\left(\rho_{-j,-k}-\frac{1}{4}\;E\right)=\frac{1}{2}\left(2I_{1j}I_{2k}\right),$$

where *j* and *k* label directions in space. In words, a two‐spin product operator is associated with a mixture of two double‐arrow states; in one of the double‐arrow states the directions are those specified in the labeling of the product operator, and in the other the directions of the arrows are reversed.

Additional examples using the generalized rules associating product operators and double‐arrow mixtures are illustrated in Figure 5.

### 5.2 Component decomposition of product operator mixtures

The arrows in the double‐arrow representation of pure states should not be confused with arrows representing vectors in semi‐classical models; they are simply indicators of a direction. One way in which they are clearly not vectors is that they do not decompose into components as classical vectors do. A naive decomposition into components of the double‐arrow state |+z;θ=π/2,ϕ⟩ (depicted on the right in Figure 2) does not work, ie,

(24) |+z;θ=π/2,ϕ⟩≠cosϕ|+z;+x⟩+sinϕ|+z;+y⟩.

**Figure 6:**
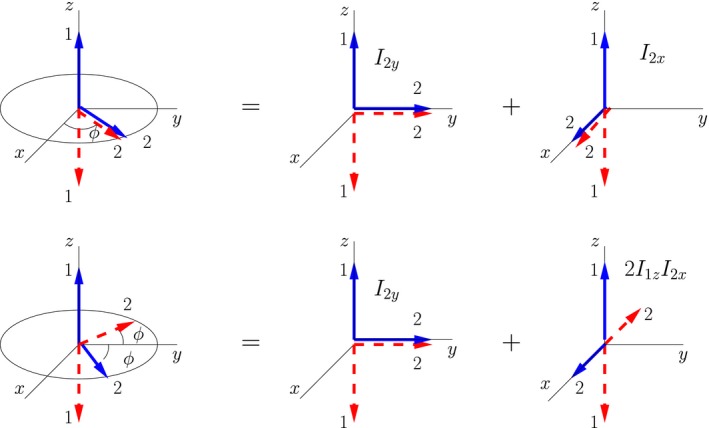
Geometric decomposition of mixtures of symmetrically oriented double‐arrow states. Weighting factors for the terms in the decomposition are given by Equations (25) and (26) [Color figure can be viewed at wileyonlinelibrary.com]

In contrast, the density matrices of some mixtures of symmetrically oriented double‐arrow states can be broken up in a component‐like manner. (A detailed discussion is contained in the Appendix.) The mixtures that “work” are those that are associated with product operators. Two examples are illustrated in Figure 6. If we combine the density matrix for the state |+z;θ=π/2,ϕ⟩ with that of the state |−z;θ=π/2,ϕ⟩, as shown in the top row of the figure (with subtraction of appropriate terms proportional to the identity matrix to make them traceless), we find

(25) $$\begin{array}{cc} & \frac{1}{2}\left(\rho_{+z,\phi }-\frac{E}{4}\right)+\frac{1}{2}\left(\rho_{-z,\phi }-\frac{E}{4}\right)\\ & =\text{cos}\phi \left[\frac{1}{2}\left(\rho_{+z,+x}-\frac{E}{4}\right)+\frac{1}{2}\left(\rho_{-z,+x}-\frac{E}{4}\right)\right]\\ & \;+\text{sin}\phi \left[\frac{1}{2}\left(\rho_{+z,+y}-\frac{E}{4}\right)+\frac{1}{2}\left(\rho_{-z,+y}-\frac{E}{4}\right)\right]\\ & =\text{cos}\phi \;I_{1x}+\text{sin}\phi \;I_{1y}. \end{array}$$

Combining the density matrix for the state |+z;θ=π/2,π/2−ϕ⟩ with that of the state |−z;θ=π/2,π/2+ϕ⟩ as shown in the bottom row of the figure (with subtraction of appropriate terms proportional to the identity matrix to make them traceless) we find

(26) $$\begin{array}{cc} & \frac{1}{2}\left(\rho_{+z,\pi /2-\phi }-\frac{E}{4}\right)+\frac{1}{2}\left(\rho_{-z,\pi /2+\phi }-\frac{E}{4}\right)\\ & =\text{cos}\phi \left[\frac{1}{2}\left(\rho_{+z,+y}-\frac{E}{4}\right)+\frac{1}{2}\left(\rho_{-z,+y}-\frac{E}{4}\right)\right]\\ & \;+\text{sin}\phi \left[\frac{1}{2}\left(\rho_{+z,+x}-\frac{E}{4}\right)+\frac{1}{2}\left(\rho_{-z,-x}-\frac{E}{4}\right)\right]\\ & =\text{cos}\phi \;I_{2y}+\text{sin}\phi \;2I_{1z}I_{2x}. \end{array}$$

The trigonometric factors in the preceding equations are not reflected in the length of any arrows in the double‐arrow representation of pure states. Rather, the trigonometric factors are simply the weights for the terms in the density matrix when it is rewritten in terms of pure states with arrows along the coordinate axes.

## 6 TIME EVOLUTION OF PRODUCT OPERATOR MIXTURES VIA PICTORIAL REPRESENTATIONS

In the preceding section I related terms in the product operator expansion with mixtures of two classically evolving double‐arrow states. To visualize the time evolution of the ensemble population corresponding to a product operator term, it is only necessary to consider two of the evolving double arrows.

### 6.1 Evolution due to external fields

**Figure 7:**
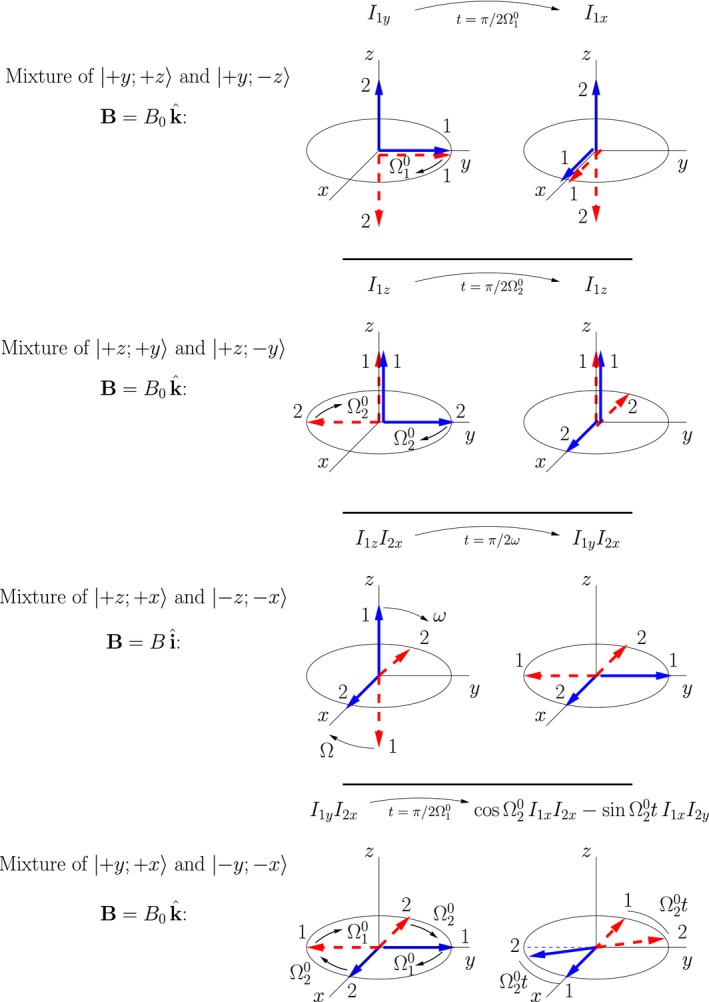
Time evolution of mixtures of double‐arrow states of spin pairs in external magnetic fields. Initial states are on the left, and states after one quarter of a precession period are on the right. Illustrations are drawn in a rotating frame

The time evolution of 50:50 mixtures of representative double‐arrow states in external magnetic fields are illustrated in Figure 7. The illustrations are drawn in the frame rotating at angular frequency ω_ref_, and the offset frequencies are given by $\Omega_{1}^{0}=\omega_{1}^{0}-\omega_{\mathrm{ref}}$ and $\Omega_{2}^{0}=\omega_{2}^{0}-\omega_{\mathrm{ref}}$. The initial states are on the left, and the states after one quarter of a relevant precession period are illustrated on the right. The associated product operators are also indicated. In the illustrated evolutions, all evolution is due to the external field, and spin‐spin coupling is assumed to be negligible. In all cases an arrow along the axis of the field is stationary, and an arrow in a plane perpendicular to the axis of the field exhibits simple precession. Notice that the external field causes single‐spin product operators to evolve into new single‐spin operators (or combinations of single‐spin operators), and two‐spin operators to evolve into two‐spin operators (or combinations of two‐spin operators). Field driven evolution of all terms in the product operator expansion can be understood with similar pictures.

Not only do the pictures Figure 7 present a very classical picture of precessing spins, they also provide simple tools for determining the full quantum density matrix of the system. Expressions for each of the quantum states represented by the double arrows are given by Equation (9), from which the density matrix is easy to calculate. [Color figure can be viewed at wileyonlinelibrary.com]

### 6.2 *J*‐coupling evolution

**Figure 8:**
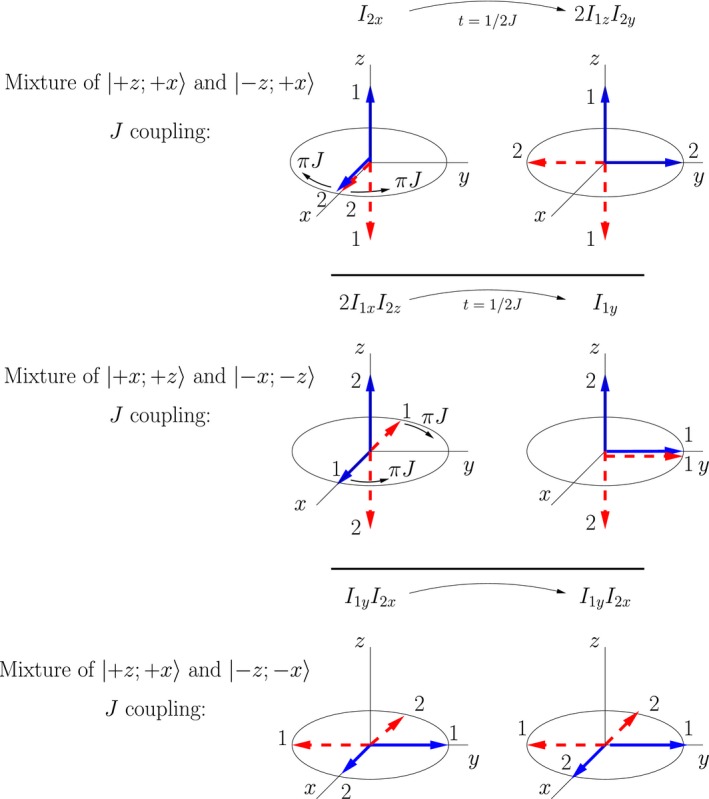
Time evolution of mixtures of double‐arrow states of spin pairs due to *J*‐coupling of the spin pairs. The top figure is drawn in a frame rotating at $\omega_{2}^{0}$, and the middle figure is in a frame rotating at $\omega_{1}^{0}$ [Color figure can be viewed at wileyonlinelibrary.com]

The time evolution of 50:50 mixtures of representative double‐arrow states due to *J*‐coupling of the spins is illustrated in Figure 8. The initial states are on the left, and the states after one quarter of a relevant precession period are illustrated on the right. The associated product operators are also indicated. The illustrated evolutions are drawn in the appropriate rotating frame so that they only include the contribution from the spin‐spin coupling. In the top illustration, the *J*‐coupling causes the spin 2 coupled to the spin‐up spin 1 (solid blue double‐arrow) to decrease its precession rate, and the spin 2 coupled to the spin‐down spin 1 (red dotted‐line double‐arrow) to increase its rate. This is an example of a single‐spin product operator evolving into a two‐spin operator, *I*_2*x*_ → 2*I*_1*z*_ *I*_2*y*_. The converse is displayed in the middle illustration in which 2*I*_1*x*_ *I*_2*z*_ → *I*_2*y*_.

The bottom illustration in Figure 8 shows the case in which both arrows in each of the spin states lie in the *x*‐*y* plane. In the top two illustrations, the precession rate of a spin in the *x*‐*y* plane depends on the “up” or “down” orientation of the other spin. In the bottom illustration the “other” spin is half way between up and down, and the net result for the orientation dependence of this state is that the density matrix of the mixture of the two double‐arrow states does not evolve in time. This assertion is discussed in more detail in the Appendix.

## 7 VISUALIZING SPIN EVOLUTION IN A COSY PULSE SEQUENCE

### 7.1 The COSY sequence

**Figure 9:**
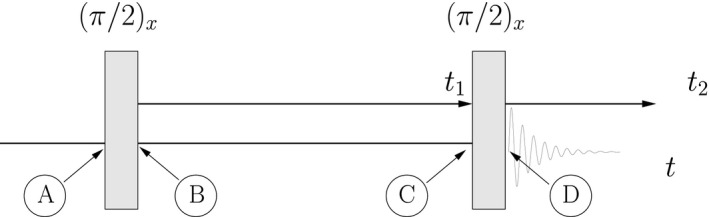
COSY pulse sequence

As a demonstration of how double‐arrow states can be used to understand the effects of pulse sequences on spin pairs, I discuss in this section the well‐known *correlation spectroscopy*, or COSY, experiment. In a COSY experiment molecules with two weakly interacting spins, like those discussed in Section 3, are subject to two π/2 pulses with a variable delay between the pulses, as illustrated in Figure 9. This experiment is analyzed using product operators in many texts (see, for example^2^). Because COSY experiments are so widely discussed elsewhere I will not dwell on the implications of such sequences in terms of experimental results of two‐dimensional spectra; I will limit myself to a brief discussion of the time evolution of the system in terms of double‐arrow states.

### 7.2 Thermal equilibrium ensemble of spin pairs

NMR experiments begin with samples in thermal equilibrium. The approximate density matrix corresponding to an ensemble of weakly interacting spin pairs in thermal equilibrium is

(27) $$\rho^{\mathrm{eq}}≃\frac{1}{4}\left(\begin{array}{cccc} 1+B & 0 & 0 & 0\\ 0 & 1 & 0 & 0\\ 0 & 0 & 1 & 0\\ 0 & 0 & 0 & 1-B \end{array}\right),$$

where,

(28) $$B\equiv \frac{\hbar \gamma B_{0}}{k_{B}T},$$

and the assumption has been made that B≪1. This is a diagonal density matrix, which suggests a mixture of states given by the diagonal elements: spin pairs that are in the state |+*z*; +*z*〉 with probability (1+B)/4, in the state |−*z*; −*z*〉 with probability (1−B)/4, and in the states |+*z*; −*z*〉 and |−*z*; +*z*〉 with equal probability 1/4.

This interpretation of this density matrix, however, is not unique—a mixture of energy eigenstates is not the only mixture that will yield the equilibrium density matrix. It is especially fruitful to rewrite the equilibrium density matrix in terms of a mixture of classically evolving double‐arrow states.

**Figure 10:**
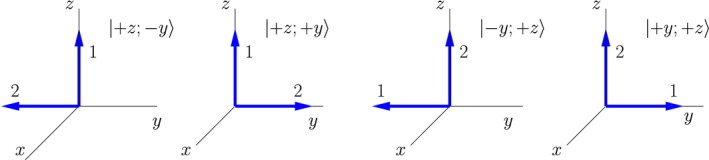
Double‐arrow states that can be used in a mixture to represent thermal equilibrium

The density matrix of Equation (27) can be written as a mixture of the double‐arrow states illustrated in Figure 10 (plus the mixture represented by the identity matrix); this mixture is

(29) $$\begin{array}{cc} \rho^{\mathrm{eq}}= & \frac{1}{4}\left(1-B\right)E+\frac{1}{4}\;B\rho_{+z,+y}+\frac{1}{4}\;B\rho_{+z,-y}+\frac{1}{4}B\;\rho_{+y,+z}\\ & +\frac{1}{4}B\;\rho_{-y,+z}, \end{array}$$

where ρ_+*z*,+*x*_ is the density matrix representation of the pure state |+*z*; +*x*〉, etc. (The first term in Equation (29) is proportional to the identity matrix, indicating a mixture of equal populations in all four conventional basis states; such a term contributes nothing to NMR observables.) It is straightforward to demonstrate the equivalence of Equations (29) and (27) by writing out the density matrices of the pure state terms directly, but for those familiar with the product operator formalism it is perhaps easier to rewrite the equilibrium density matrix as

(30) $$\rho^{\mathrm{eq}}=\frac{1}{4}\;E+\frac{1}{4}\;I_{1z}+\frac{1}{4}\;I_{2z},$$

and then use Equations (17a) and (17b) to rewrite ρ^eq^ in terms of the density matrices of pure double‐arrow states.

The specific mixture given in Equations (29) and (30) is not the only way to rewrite the equilibrium density matrix, and it is reasonable to ask why this would be a good starting point. In the following section I analyze a multiple‐pulse NMR experiment, and demonstrate that this initial state leads to a sequence of classically evolving double‐arrow states. As will be illustrated in the following section in the analysis of an experiment with a sequence of rf pulses, this choice leads to a sequence of the special‐case double arrow states with classical time evolution. [Color figure can be viewed at wileyonlinelibrary.com]

### 7.3 Evolution of double‐arrow states in a COSY sequence

In this section I illustrate the time evolution of the mixture of double‐arrow states |+*z*; +*y*〉 and |+*z*, −*y*〉 in a COSY sequence; these are the pure double‐arrow states associated with the product operator *I*_1*z*_. (The evolution of the mixture of |+*y*; +*z*〉 and | −*y*, +*z*〉 associated with *I*_2*z*_ is identical, save for a swap of indices.) For ease of visualization I will work in a frame rotating with a frequency $\omega_{1}^{0}$, ie, in a frame in which there is zero offset from the precession frequency of an isolated nucleus 1. Generalization to an arbitrary offset requires additional geometric analysis, but no additional physics.

**Figure 11:**
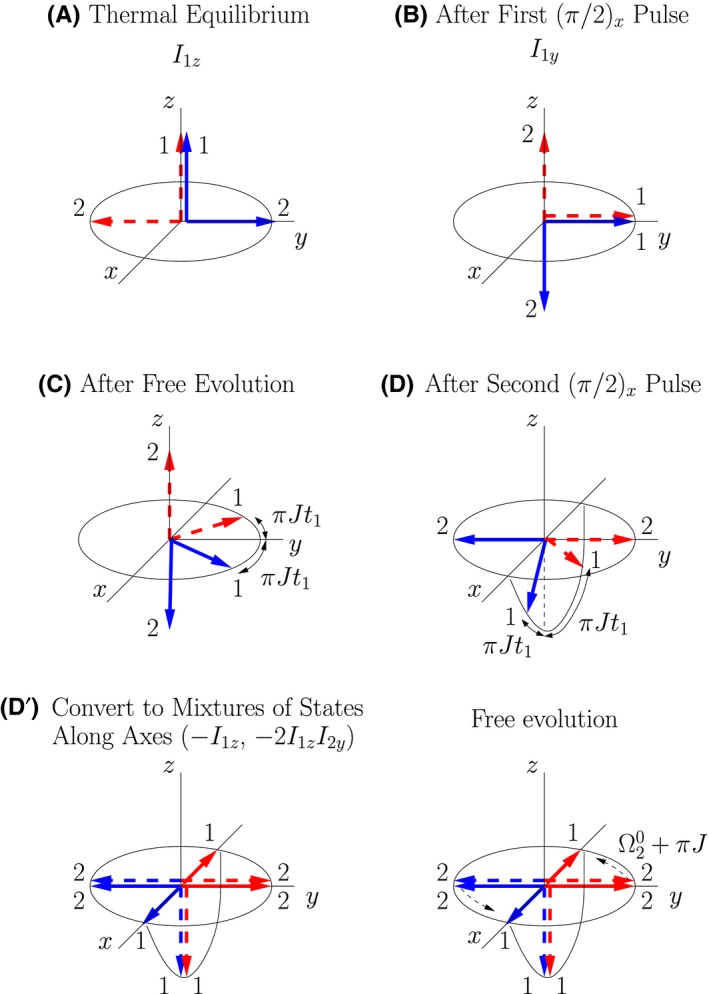
Representation of the COSY time evolution of the mixture corresponding to the *I*_1*z*_ term in the product operator expansion [Color figure can be viewed at wileyonlinelibrary.com]

The time evolution of the double‐arrow states is illustrated in Figure 11. The letters correspond to various points in the pulse sequence illustrated in Figure 9. The thermal equilibrium mixture of |+*z*; +*y*〉 and |+*z*, −*y*〉 is illustrated in Part **A** of the figure. The first (π/2)_*x*_ pulse rotates all arrows clockwise around the *x*‐axis, resulting in both arrows for nucleus 1 pointing in the +*y* direction, and the arrows of nucleus 2 pointing in the +*z* and −*z* directions as is illustrated in Part **B**. This result is another mixture of two double‐arrow states that both have simple classical‐like time evolution.

The thermal equilibrium density matrix could have been written in terms of a mixture of |+*z*; +*x*〉 and |+*z*, −*x*〉, but the result of the first rf pulse would have been a mixture of double‐arrow states with both vectors in the plane perpendicular to the field. Such states do not exhibit simple classical evolution or quantum evolution (see the Appendix for more detailed analysis of the quantum evolution). In this sense, classical intuition can be used as a guide to the selection of mixtures of quantum states that will be easy to analyze. The results using the second version of the equilibrium density matrix would not be wrong; they just would not be as easy to analyze.

During the period of free evolution between the rf pulses, the dotted‐line red arrow of nucleus 1 precesses at a lab frequency of magnitude $\omega_{1}^{0}-\pi J$, because it represents a pure state of nucleus 1 with a spin‐up nucleus 2, and the solid blue arrow arrow of nucleus 1 precesses at a lab frequency of magnitude $\omega_{1}^{0}+\pi J$, because it represents a pure state of nucleus 1 with a spin‐down nucleus 2. In the frame rotating with frequency $\omega_{1}^{0}$, these arrows move apart, with individual frequencies ±π*J*. At time time *t*_1_ (**C**) they make angles ±π*Jt*_1_ with respect to the *y*‐axis. The arrows for nucleus 2 represent stationary states, so they do not evolve during this period. The second (π/2)_*x*_ pulse once again rotates all arrows clockwise in cones around the *x*‐axis. This brings the arrows for nucleus 2 to the +*y* and −*y* axes, and it rotates the arrows for nucleus 1 into the *x*‐*z* plane. The arrows for nucleus 1 now form angles of ±π*Jt*_1_ with respect to the −*y*‐axis.

At point **D** after the second rf pulse, the system is in a mixture of double‐arrow states that do not evolve in a simple way. (Note that interacting classical moments with the same orientations do not exhibit simple precession either.) At this point it is necessary to decompose the mixture into a new mixture of states that do evolve in simple ways, as is discussed in Section 5.2. In terms of pure states, we can decompose the mixture of double‐arrow states labeled **D** in Figure 11 into the mixture labeled **D** ^′^. In terms of product operators, the mixture of the blue dotted‐line double‐arrow state and the red dotted‐line double‐arrow state corresponds to the product operator −*I*_1*z*_, and the mixture of the solid blue double‐arrow state and the solid red double‐arrow state corresponds to the product operator −2*I*_1*z*_ *I*_2*y*_. The relative contributions of the dotted‐line states and the solid‐line states in the decomposition are determined by the techniques of Section 5.2, and given by the geometry in part **D** of the figure:

(31) $$\rho_{D^{\prime }}=-\text{cos}\left(\pi Jt_{1}\right)\;I_{1z}-\text{sin}\left(\pi Jt_{1}\right)\;2I_{1x}I_{2y}.$$

During the period of free evolution after **D** we can use the results discussed in Section 6. The double‐arrow states corresponding to −*I*_1*z*_ (drawn with dotted lines) rotate clockwise at $\Omega_{2}^{0}+\pi J$, and the double arrows corresponding to −2*I*_1*x*_ *I*_2*y*_ do not precess.

If the offset of nucleus 1, $\Omega_{1}^{0}$, is not zero, the geometry of the arrows in illustrations like those in Figure 11 becomes more complicated. The arrows of nucleus 1 are not separated symmetrically at **C** and **D**, meaning that the decomposition resulting in the mixture of states represented in **D** ^′^ will have more states in it, corresponding to additional product operator terms. This complication introduces no new physics.

## 8 CONCLUSION

A complete understanding of the behavior of a microscopic system, like a molecule with coupled spins, requires quantum mechanics. This fact does not preclude, however, a quantum system from evolving in some instances in a way that parallels that of a system governed by the laws of classical mechanics. In some systems the classical‐quantum parallels are qualitative; in the time evolution of the quantum double‐arrow states of coupled spins (and mixtures of these states) that I have discussed in this paper, the parallels can be quantitative and complete. By complete, I mean that given an initial quantum description of an ensemble, the final quantum description of the ensemble can be determined solely using a classical analog of the system. The existence of a classical analog for ensembles of weakly coupled spins can help guide the intuition of the NMR expert sophisticated in the use of quantum mechanics. For the novice at the other end of the spectrum of expertise, the classical analog provides a straightforward way to visualize the time evolution of correlated spins that is free of the quantum machinery of product operators, unitary transformations of operators, and commutation relations. (The pedagogical challenge becomes one of justifying to the novice the initial state as a representation of thermal equilibrium.) The classical evolution offers the potential to introduce the novice to multiple‐pulse NMR spectroscopy by focusing on concepts, while leaving the development of quantum mechanical tools to a later time.

## APPENDIX (A1)

### QUANTUM MECHANICS OF PRECESSION

#### Precession of single spins

The quantum state |θ, φ〉 of a single spin represented by an “arrow” oriented in the direction given by the angles θ and φ was detailed in Equation (3). This state is a linear combination of eigenstates of the Hamiltonian for a magnetic moment in a field in the *z* direction. The time evolution of such eigenstates is simple: the time dependence of each eigenstate is contained in a multiplicative phase factor, $e^{-iE_{j}t/\hbar }$, where *E*_*j*_ is the energy of the eigenstate. Thus, for a moment in a magnetic field, the initial state |ψ(0)〉 = |θ, φ〉 evolves into the state

(A1) $$\begin{array}{cc} \left|\psi (t)\right\rangle & =\text{cos}\frac{\theta }{2}\;e^{+i\omega^{0}t/2}\left|+z\right\rangle +e^{i\phi }\text{sin}\frac{\theta }{2}\;e^{-i\omega^{0}t/2}\left|-z\right\rangle \\ & =e^{i\omega_{0}t/2}\left(\text{cos}\frac{\theta }{2}\;|+z\rangle +\text{sin}\frac{\theta }{2}\;e^{i(\phi -i\omega_{0}t)}\left|-z\right\rangle \right)\\ & =e^{i\omega^{0}t/2}\left|\theta ,\phi -\omega^{0}t\right\rangle \\ & ⟶e^{i\omega^{0}t/2}\left(\begin{array}{c} \text{cos}\frac{\theta }{2}\\ e^{i(\phi -\omega^{0}t)}\text{sin}\frac{\theta }{2} \end{array}\right). \end{array}$$

This time‐dependent state corresponds to an arrow state precessing at a rate ω^0^ = γ*B*_0_ at a constant inclination angle θ with respect to the direction of the field. The azimuthal angle decreases with time, corresponding to precession in a clockwise direction, as viewed from the +*z* direction. (The overall phase factor $e^{i\omega^{0}t/2}$ is inconsequential, and disappears in the density matrix representation of the state. This phase has nothing to do with the phase angle of precession.)

#### Precession of spin pairs

##### Quantum evolution of general double‐arrow spin‐pair states

The general form of a double‐arrow state is given in Equation (9). As in the case of a single spin, this is a linear combination of energy eigenstates, and again it is straightforward to determine the time evolution using multiplicative phase factors for each energy eigenstate in the combination. An initial double‐arrow state |ψ(0)〉 = |θ_*A*_, φ_*A*_; θ_*B*_, φ_*B*_〉 evolves into the state

(A2) $$\begin{array}{cc} \left|\psi (t)\right\rangle & =\left(\text{cos}\frac{\theta_{A}}{2}\text{cos}\frac{\theta_{B}}{2}\right)e^{-i(-\omega_{A}^{0}-\omega_{B}^{0}+\pi J)t/2}\left|+z;+z\right\rangle \\ & \;+\left(e^{i\phi_{B}}\text{cos}\frac{\theta_{A}}{2}\text{sin}\frac{\theta_{B}}{2}\right)e^{-i(-\omega_{A}^{0}+\omega_{B}^{0}-\pi J)t/2}\left|+z;-z\right\rangle \\ & \;+\left(e^{i\phi_{A}}\text{sin}\frac{\theta_{A}}{2}\text{cos}\frac{\theta_{B}}{2}\right)e^{-i(\omega_{A}^{0}-\omega_{B}^{0}-\pi J)t/2}\left|-z;+z\right\rangle \\ & \;+\left(e^{i(\phi_{A}+\phi_{B})}\text{sin}\frac{\theta_{A}}{2}\text{sin}\frac{\theta_{B}}{2}\right)e^{-i(\omega_{A}^{0}+\omega_{B}^{0}+\pi J)t/2}\left|-z;-z\right\rangle \\ & =e^{i(\omega_{A}^{0}+\omega_{B}^{0}-\pi J)t/2}\left[\left(\text{cos}\frac{\theta_{A}}{2}\text{cos}\frac{\theta_{B}}{2}\right)\left|+z;+z\right\rangle \right.\\ & \left.\;+\left(\text{cos}\frac{\theta_{A}}{2}\text{sin}\frac{\theta_{B}}{2}\right)e^{i[\left(\right.\phi_{B}-\left(\omega_{B}^{0}-\pi J\right)t]}\;\left|+z;-z\right\rangle \right.\\ & \left.\;+\left(\text{sin}\frac{\theta_{A}}{2}\text{cos}\frac{\theta_{B}}{2}\right)e^{i[\phi_{A}-\left(\omega_{A}^{0}-\pi J\right)t]}\;\left|-z;+z\right\rangle \right.\\ & \left.\;+\left(\text{sin}\frac{\theta_{A}}{2}\text{sin}\frac{\theta_{B}}{2}\right)e^{i(\phi_{A}-\omega_{A}^{0}+\phi_{B}-\omega_{B}^{0}t)}\;\left|-z;-z\right\rangle \right]\\ & \;⟶e^{i(\omega_{B}^{0}+\omega_{B}^{0}-\pi J)t/2}\left(\begin{array}{c} \text{cos}\frac{\theta_{A}}{2}\text{cos}\frac{\theta_{B}}{2}\\ e^{i[\phi_{B}-\left(\omega_{B}^{0}-\pi J\right)t]}\text{cos}\frac{\theta_{A}}{2}\text{sin}\frac{\theta_{B}}{2}\\ e^{i[\phi_{A}-\left(\omega_{A}^{0}-\pi J\right)t]}\text{sin}\frac{\theta_{A}}{2}\text{cos}\frac{\theta_{B}}{2}\\ e^{i(\phi_{A}-\omega_{A}^{0}t+\phi_{B}-\omega_{B}^{0}t)}\text{sin}\frac{\theta_{A}}{2}\text{sin}\frac{\theta_{B}}{2} \end{array}\right). \end{array}$$

##### Special Case I: Quantum evolution of uncoupled spin‐pair states

When the spins are not coupled, ie, when *J* = 0, the initial product state evolves to

(A3) $$\begin{array}{cc} {\left|\psi \left(t\right)\right\rangle }_{J=0} & =e^{i(\omega_{A}^{0}+\omega_{B}^{0})t/2}\left[\left(\text{cos}\frac{\theta_{A}}{2}\text{cos}\frac{\theta_{B}}{2}\right)\left|+z;+z\right\rangle \right.\\ & \left.\;\;\;\;+\left(\text{cos}\frac{\theta_{A}}{2}\text{sin}\frac{\theta_{B}}{2}\right)e^{i(\phi_{B}-\omega_{B}^{0}t)}\;\left|+z;-z\right\rangle \right.\\ & \left.\;\;\;\;+\left(\text{sin}\frac{\theta_{A}}{2}\text{cos}\frac{\theta_{B}}{2}\right)e^{i}\left[\left(\phi_{A}-\omega_{A}^{0}t\right)\right]\;\left|-z;+z\right\rangle \right.\\ & \left.\;\;\;\;\;+\left(\text{sin}\frac{\theta_{A}}{2}\text{sin}\frac{\theta_{B}}{2}\right)e^{i(\phi_{A}-\omega_{A}^{0}t+\phi_{B}-\omega_{B}^{0}t)}\;\left|-z;-z\right\rangle \right]\\ & =e^{i(\omega_{A}^{0}+\omega_{B}^{0})t/2}\left(\text{cos}\frac{\theta_{A}}{2}{\left|+z\right\rangle }_{A}+e^{i(\phi_{A}-\omega_{A}^{0}t)}\text{sin}\frac{\theta_{B}}{2}{\left|-z\right\rangle }_{A}\right)\\ & \;\;\;\;⊗\left(\text{cos}\frac{\theta_{B}}{2}{\left|+z\right\rangle }_{B}+e^{i(\phi_{B}-\omega_{B}^{0}t)}\text{sin}\frac{\theta_{B}}{2}{\left|-z\right\rangle }_{B}\right), \end{array}$$

which is the direct product of two independently precessing single‐arrow states. This shows that the two arrows of a double‐arrow quantum state precess independently at their classical frequencies in the absence of spin‐spin coupling.

##### Special Case II: Coupled spin pairs—One arrow along direction of field

When the spins are coupled, the general result factors into independent single‐arrow states if one of the single‐arrow states lies along the *z*‐axis, ie, θ_*A*_ = 0, or θ_*A*_ = π, or θ_*B*_ = 0, or θ_*B*_ = π. When θ_*A*_ = 0, the general time‐dependent state becomes

(A4) $$\begin{array}{cc} {\left|\psi \left(t\right)\right\rangle }_{\theta_{A}=0} & =\;e^{i(\omega_{A}^{0}+\omega_{B}^{0}-\pi J)t/2}\\ & \;\;\left(\text{cos}\frac{\theta_{B}}{2}\;|+z;+z\rangle +\text{sin}\frac{\theta_{B}}{2}\;e^{i[\left(\right.\phi_{B}-\left(\omega_{B}^{0}-\pi J\right)t]}\;\left|+z;-z\right\rangle \right)\\ & =\;e^{i(\omega_{A}^{0}+\omega_{B}^{0}-\pi J)t/2}\;{\left|+z\right\rangle }_{A}\\ & \;\;⊗\left(\text{cos}\frac{\theta_{B}|}{2}{\left|+z\right\rangle }_{B}+e^{i[\phi_{A}-\left(\omega_{B}^{0}-\pi J\right)t]}\text{sin}\frac{\theta_{B}}{2}{\left|-z\right\rangle }_{B}\right). \end{array}$$

This is the direct product of a stationary single‐arrow state oriented in the +*z* direction, and a single‐arrow state precessing with an angular frequency $\omega_{B}^{0}-\pi J$ (and again there is an inconsequential overall phase factor). For the state with θ_*A*_ = π, similar analysis shows that spin B precesses with a frequency $\omega_{B}^{0}+\pi J$. Analogous results hold for θ_*B*_ = 0 and θ_*B*_ = π.

##### Special Case III: Coupled Spins—both arrows perpendicular to direction of magnetic field

When coupled spins are in a state with both arrows oriented perpendicular to the direction of the magnetic field, ie, θ_*A*_ = θ_*B*_ = π/2, the general time‐dependent state given in Equation (A2) reduces to

(A5) $$\begin{array}{cc} {\left|\psi \left(t\right)\right\rangle }_{\theta_{A}=\theta_{B}=\pi /2}= & \frac{1}{2}e^{i(\omega_{A}^{0}+\omega_{B}^{0})t/2}\\ & \left[\left|+z;+z\right\rangle +e^{i[\phi_{B}-\left(\omega_{B}^{0}-\pi J\right)t]}\;\left|;+z;-z\right\rangle \right.\\ & \left.+e^{i[\phi_{A}-\left(\omega_{A}^{0}-\pi J\right)t]}\;\left|-z;+z\right\rangle \right.\\ & \left.+e^{i(\phi_{A}-\omega_{A}^{0}t+\phi_{B}-\omega_{B}^{0}t)}\;\left|-z;-z\right\rangle \right]. \end{array}$$

This state does *not* factor into two single‐arrow states exhibiting simple precession. For example, the spins may start in the factored state |ψ(0)〉 = |+*x*; +*y*〉, but they do not evolve into a state that can be factored, ie,

(A6) $$\left|\psi \left(t\right)\right\rangle \neq {|\theta_{1}\;=\;\pi /2\;;\;\phi_{1}\;=\;-\omega_{1}^{0}t〉}_{1}⊗{|\theta_{2}\;=\;\pi /2\;;\;\phi_{2}\;=\;\pi /2-\omega_{2}^{0}t〉}_{2}.$$

However, a mixture of two symmetrically oriented states gives a result that does exhibit simple precession. For example, the terms in the density matrix for a mixture of the pure states |+*x*; +*y*〉 and |−*x*; −*y*〉 do factor. If we write time‐dependent density matrix for the pure double‐arrow state that starts in |+*x*;+*y*〉 as ρ_+*x*,+*y*_(*t*), etc., we find

(A7) $$\begin{array}{cc} & \frac{1}{2}\left[\rho_{+x,+y}\left(t\right)+\rho_{-x,-y}\left(t\right)\right]\\ & =\frac{1}{4}\left(\begin{array}{cccc} 1 & 0 & 0 & -ie^{i(\omega_{1}^{0}+\omega_{2}^{0})t}\\ 0 & 1 & ie^{i(\omega_{1}^{0}-\omega_{2}^{0})t} & 0\\ 0 & -ie^{i(-\omega_{1}^{0}+\omega_{2}^{0})t} & 1 & 0\\ +ie^{-i(\omega_{1}^{0}+\omega_{2}^{0})t} & 0 & 0 & 1 \end{array}\right). \end{array}$$

Notice that the result does not contain *J*, the spin coupling constant. Comparing this to the density matrix of a mixture of independent spins (ie, without any coupling), we find the same result, ie,

(A8) $$\begin{array}{cc} & \frac{1}{2}\left[\rho_{+x}^{\left(1\right)}\left(t\right)⊗\rho_{+y}^{\left(2\right)}\left(t\right)+\rho_{-x}^{\left(1\right)}\left(t\right)⊗\rho_{-y}^{\left(2\right)}\left(t\right)\right]\\ & \;=\frac{1}{2}\left[\rho_{+x,+y}\left(t\right)+\rho_{-x,-y}\left(t\right)\right], \end{array}$$

where $\rho_{+x}^{\left(1\right)}\left(t\right)$ is the time‐dependent density matrix for a single spin initially oriented in the +*x* direction, and precessing with angular frequency $\omega_{1}^{0}$, etc. The equivalence given in Equation (A8) justifies using the classical free evolution of the arrows in double‐arrow states when both arrows are in the *x*‐*y* plane, as long as the states are in a 50:50 mixture of states with oppositely oriented arrows. (This is equivalent to saying that the product operator term 2*I*_1*x*_ *I*_2*y*_ does not evolve under *J* coupling.)
